# A Complete Approach for circRNA Therapeutics from Purification to Lyophilized Delivery Using Novel Ionizable Lipids

**DOI:** 10.3390/ijms26115138

**Published:** 2025-05-27

**Authors:** Esther Broset, Ana Larraga, Verónica Lampaya, Víctor Navarro, Alexandre López-Gavín, Diego de Miguel, Álvaro Peña, Juan Martínez-Oliván, Diego Casabona

**Affiliations:** Certest Pharma, Certest Biotec Ltd., Polígono Industrial Río Gallego II, Calle J, 1, 50840 San Mateo de Gállego, Spain; ebroset@certest.es (E.B.); alopez@certest.es (A.L.-G.); dmiguel@certest.es (D.d.M.); apena@certest.es (Á.P.)

**Keywords:** LNP, circRNA, ionizable lipids, Lyophilization, scalable purification

## Abstract

Circular RNA (circRNA) has gained significant attention as a potential therapeutic tool due to its remarkable stability and resistance to degradation by exonucleases. However, scalable and efficient methods for purification and delivery remain critical challenges that must be addressed. In this study, we developed and evaluated an optimized affinity chromatography method using Oligo (dT) columns for the purification of circRNA, achieving high yield and purity compared with high-performance liquid chromatography. Additionally, we investigated the in vivo efficacy of circRNA-Oligo (dT) encapsulated in lipid nanoparticles (LNPs) formulated with emerging ionizable lipids, including CP-LC-0867 and CP-LC-0729. Our results showed that LNPs formulated with CP-LC-0867 consistently produced higher protein expression compared to SM-102, with sustained luciferase activity observed over a 14-day period. Furthermore, we assessed the lyophilization potential of LNP-circRNA-Oligo (dT) using CP-LC-0729 to extend the shelf life and eliminate the need for ultra-low-temperature storage. Remarkably, the lyophilized LNPs exhibited no significant differences in protein expression compared to their non-lyophilized counterparts, demonstrating that lyophilization is a viable strategy for extending the storage and transport of circRNA therapies. These findings underscore the potential of optimized new ionizable lipids, improved purification strategies, and lyophilization techniques to enhance the scalability, stability, and practical application of circRNA therapies.

## 1. Introduction

Circular RNA (circRNA) has emerged as a novel and versatile class of RNA molecules, distinguished by their covalently closed loop structure. This topology grants circRNA remarkable stability, as it lacks the 5′ and 3′ ends that make linear RNA susceptible to exonuclease-mediated degradation. Furthermore, the use of the internal ribosome entry site (IRES) in circRNAs allows them to be translated [1], which has increased the attention they have received in various fields, including gene therapy [2], vaccine development [3,4], and synthetic biology [5]. Its ability to sustain prolonged protein expression makes circRNA an attractive alternative to traditional linear RNA [6], particularly in therapeutic applications where long-lasting effects are desired without the risks associated with genomic integration.

The production of circRNA typically involves in vitro transcription (IVT) with a subsequent circularization and a complex purification, a process to separate circular from linear RNA species [7]. The circularization process has recently evolve from the use of low effective T4 ligase-mediated reactions [8] to the utilization of the highly efficient engineered permuted intron–exon (PIE) self-splicing strategy to promote the circularization of RNA molecules [9,10]. However, the efficient purification of circRNA from precursor, introns, and nicked RNA remains a significant challenge. Those RNA contaminants can compromise the immunity and functionality of circRNA [11,12], which is particularly problematic in applications that require high fidelity and minimal interference, such as in RNA vaccines and gene therapies [13]. Traditional methods for RNA purification, such as gel electrophoresis [5] and high-performance liquid chromatography (HPLC) [9], have been used to isolate circRNA. Nevertheless, RNA yields from these methods are typically less than 1% of the input RNA [2,9], making large-scale circRNA production economically challenging.

The lipid nanoparticle (LNP) system is one of the more effective for in vivo delivery of circRNA-based therapies. LNPs are typically composed of four key components: ionizable lipids, phospholipids, cholesterol, and polyethylene glycol (PEG)-lipids. Among these, ionizable lipids are crucial for the efficient release of RNA cargo into the cytoplasm. These ionizable lipids acquire a positive charge in acidic environments, such as within endosomes, facilitating the release of RNA into the cytoplasm. Despite their pivotal role, current ionizable lipids present several limitations that hinder their broader application. Firstly, endosomal escape is highly inefficient, with only 1–2% of internalized LNPs successfully releasing their RNA cargo into the cytoplasm [14]. This represents a major bottleneck in the intracellular delivery process, significantly limiting the therapeutic potency of RNA-based interventions [15]. Additionally, the vast majority of ionizable lipid screenings have been conducted using mRNA as the model cargo, resulting in limited data on the performance and functionality of LNPs when encapsulating alternative RNA modalities, such as self-amplifying RNA or circRNA [16]. Toxicity is another concern; some ionizable lipids have been associated with elevated inflammatory responses [17]. Moreover, the limited number of clinically approved ionizable lipids, such as MC3, SM-102, and ALC-0315, poses challenges related to complex synthesis process [18] and licensing restrictions and high costs, thereby complicating access and increasing the expense of RNA-based treatments [19]. In this context, the development of novel lipid formulations that are accessible at an affordable cost is not only scientifically necessary but also critical from a public health and equity standpoint [20].

Additionally to the lipid components, the physical and chemical stability of LNP formulations represent another significant challenge for their widespread application [21]. Current RNA-LNP therapies, including the approved mRNA vaccines Comirnaty and Spikevax, require cryogenic preservation and transportation between −80 °C and −60 °C for Comirnaty and at −20 °C for Spikevax [22]. The stringent storage requirements are primarily due to the intrinsic sensitivity of mRNA to environmental factors such as oxygen, enzymatic degradation, and fluctuations in pH. Additionally, complex interactions between the lipid components and degradation by-products from oxidation and hydrolysis further compromise the stability of mRNA. As water, oxygen, and lipids are included in mRNA-LNP formulations, ensuring high stability in liquid mRNA-LNP systems has historically presented some difficulties [23].

Enhancing the stability of mRNA-LNP therapies has shown promise through lyophilization, a process that removes water by sublimation under vacuum at low temperatures [24,25]. One study reported successful lyophilization of mannose-modified LNPs containing SM-102 as the ionizable lipid, resulting in a durable immune response against rabies and SARS-CoV-2 viruses [26]. While lyophilization has been primarily developed for mRNA-based LNPs, there is limited research on its application to circRNA-LNP formulations. Consequently, developing a circRNA-LNP platform that incorporates novel ionizable lipids capable of withstanding the lyophilization process will be crucial for making these therapies more accessible and practical for public health applications.

In this study, we introduce a complete circRNA platform designed to overcome the economic and industrial challenges associated with circRNA-LNP-based therapies. To address the critical issue of low purification yields, we propose an innovative purification method utilizing poly(A) affinity chromatography. This approach significantly enhances purification efficiency, achieving yields comparable to those of mRNA, thus rendering the process feasible for industrial-scale production. Furthermore, we address the challenge of ionizable lipid selection, a key component in LNP formulations, by exploring novel lipid candidates’ performance with circRNA [27,28]. Finally, we integrate a lyophilization technique tailored to circRNA-LNP formulations [29], ensuring the practicality and accessibility of these therapies for widespread health applications.

## 2. Results

### 2.1. Optimization of In Vitro Transcription Conditions for Enhanced CircRna Production

To optimize our circRNA production platform, we utilized the PIE self-splicing circularization system, which is widely recognized for its efficiency [9]. The initial focus was on enhancing in vitro transcription (IVT) conditions, particularly incubation time and Mg^2+^ concentration, as both are critical determinants of yield and RNA quality [30]. For circRNA production, the Mg^2+^ concentration is particularly important, as it is essential not only for the IVT process but also for the PIE-mediated circularization.

**Figure 1:**
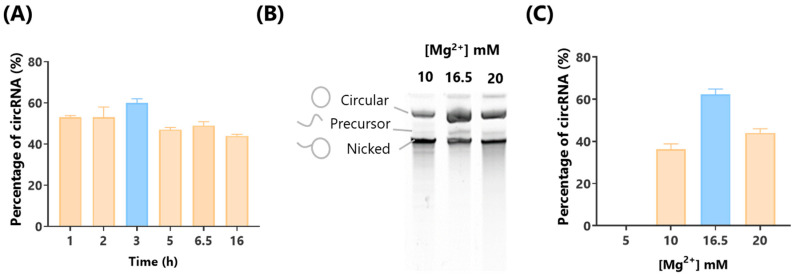
Optimization of incubation time and Mg^2+^ concentration for circRNA production. (**A**) Quantification of circular isoform yield at 1, 2, 3, 5, 6.5, and 16 h of incubation during the in vitro transcription (IVT) reaction. (**B**) Polyacrylamide gel electrophoresis (PAGE) analysis of circRNA yield at different Mg^2+^ concentrations (10, 16.5, and 20 mM) used on the IVT reaction buffer. (**C**) Percentage of circular RNA isoform based on PAGE results from panel (**B**) quantified by image analysis.

Our optimization began by adjusting the incubation time while maintaining a Mg^2+^ concentration of 16.5 mM and an incubation temperature of 37 °C. We observed that a 3 h incubation period resulted in the highest percentage of circRNA isoforms, indicating it as the optimal duration (Figure 1A). We next varied the Mg^2+^ concentration to evaluate its effect on circRNA yield. In line with our previous optimization studies for mRNA IVT [31], we identified 16.5 mM of Mg^2+^ as the optimal concentration for circRNA production (Figure 1B,C). Under these conditions, approximately 60% of the RNA was in the circular isoform, compared to 40% observed with 20 mM and 10 mM of Mg^2+^. To quantify the proportion of circular isoforms, we performed PAGE electrophoresis coupled with image analysis (Figure 1B) and confirmed the results using capillary electrophoresis, which yielded consistent findings (Figure S1). Notably, reducing the Mg^2+^ concentration from 10 mM to 5 mM resulted in a complete absence of RNA synthesis. This underscores the critical role of Mg^2+^ concentration in the formation of both mRNA and circRNA.

### 2.2. Scalable circRNA Purification Using Affinity Chromatography

**Figure 2:**
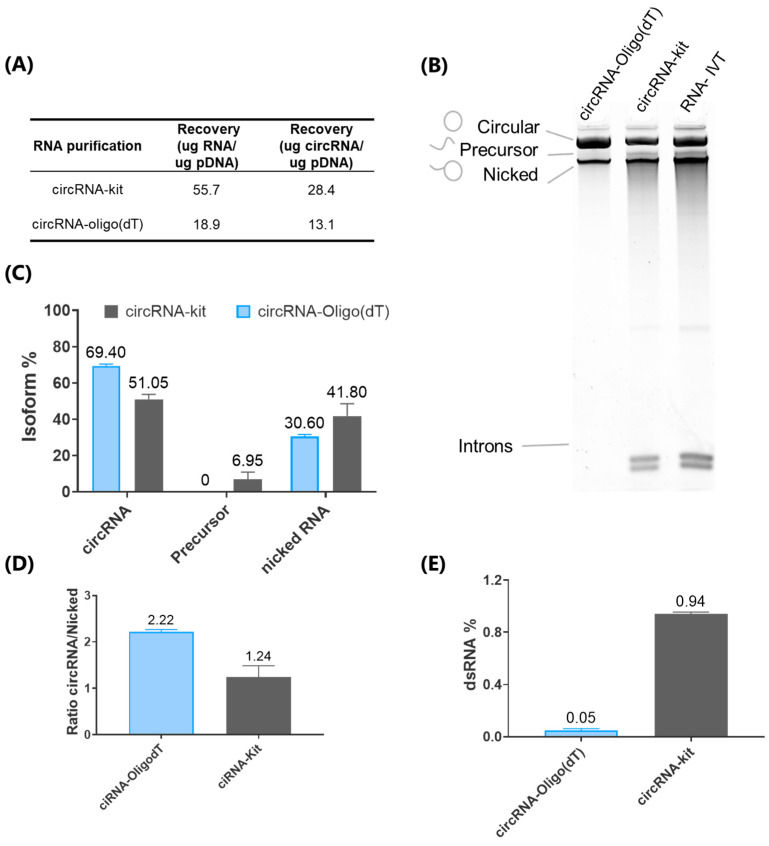
Comparison of circRNA Purification Methods and Quality Assessment. (**A**) Total RNA and circRNA recovery per µg of in vitro transcription (IVT) reaction input pDNA for Oligo (dT) affinity chromatography compared to the silica-based circRNA-kit method. (**B**) Denaturing polyacrylamide gel electrophoresis (PAGE) analysis of RNA before purification (RNA-IVT) and post-purification using either the silica or Oligo (dT) methods. Circular RNA, precursor, nicked, and intronic RNA species are indicated. (**C**) Quantification of the percentage of circular RNA, precursor, and nicked isoforms based on PAGE results in panel (**B**), determined through image analysis. (**D**) circRNA/nicked RNA ratio from panel (**C**). (**E**) Quantification of double-stranded RNA percentage (dsRNA) content through dot-blot analysis, with image-based quantification on samples purified by silica kit (circRNA-kit) or Oligo (dT) methods (circRNA-Oligo (dT).

After optimizing the IVT conditions, we shifted our focus to the purification steps. Traditionally, circRNA production involves an IVT step, followed by circularization with the addition of GTP and Mg^2+^, and a subsequent purification step. The resulting circRNA is then treated with RNase R to eliminate the linear RNA, and then it is further purified. Firstly, to maximize yield, we chose to bypass the RNase R treatment and the subsequent purification step, directly purifying the circRNA after the circularization step. Secondly, to improve purity, we leveraged the polyA tail in the circRNA design [4,9], utilizing affinity chromatography with Oligo (dT) 25 columns (Oligo (dT)) for the purification (Figure S2). This method was compared to a gold-standard approach commonly used, which utilizes silica-based purification. The Oligo (dT) method yielded an average recovery of 18.9 µg of total RNA per µg of IVT input pDNA (Figure 2A). Although this recovery rate is lower than the 55.75 µg per µg of pDNA achieved with the silica-based method (circRNA-kit), it is important to highlight that the Oligo (dT) column resulted in higher purity of the circular isoform. Specifically, the Oligo (dT) method enriched the circRNA isoform to approximately 69.4% of the total RNA, whereas the silica method reached only 51.25%, indicating a markedly better purification performance.

Additionally, after the Oligo (dT) purification, we observed the complete removal of the intron isoform and linear precursors (Figure 2B,C) that are reported to profoundly diminished translation efficiency by inducing immune activation [13]. This efficient precursor removal justifies omitting the RNase R treatment, thereby eliminating the need for an additional, costly enzymatic step. In contrast, when using the silica-based purification method, intron isoforms and linear precursors remained at levels comparable to those observed immediately after the IVT reaction. Furthermore, when evaluating purity, we found that the circRNA/nicked RNA ratio serves as a reliable purity indicator. Oligo (dT) purification achieved a two-fold higher circRNA/nicked RNA ratio compared to the silica method (Figure 2D).

The presence of dsRNA in RNA therapies has been shown to be detrimental to protein production [32,33], as it activates the innate cellular response, leading to the elimination of foreign RNA [34]. HPLC purification has been demonstrated to eliminate immune activation in IVT mRNA samples [32] and circRNA [9,13]; however, there are no data regarding its effect when using Oligo (dT) purification for circRNA. Therefore, to further assess the quality of the purified circRNA, we quantified the percentage of dsRNA% in the samples (Figure 2E). Our analysis demonstrated that affinity chromatography reduced dsRNA levels to 0.05%, significantly below the 0.5% threshold generally considered acceptable for RNA-based therapies. Notably, compared to the circRNA-kit purified using a silica-based method, our circRNA-Oligo (dT) purification resulted in an 18.8-fold reduction in dsRNA content. This substantial decrease emphasizes the capability of Oligo (dT) purification in minimizing dsRNA impurities, which is crucial for enhancing the safety and efficacy of circRNA-based therapies.

### 2.3. Oligo (dT)-Purified circRNA Exhibits Enhanced In Vitro and In Vivo Translation

**Figure 3:**
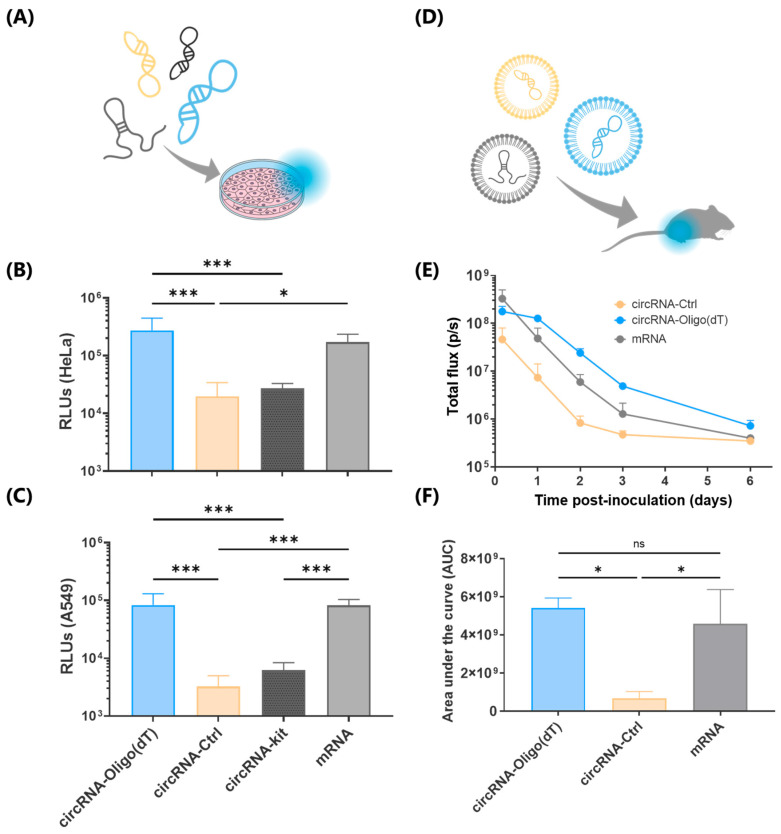
Translation efficacy of circRNA purified by Oligo (dT) affinity chromatography. (**A**) Schematic of in vitro transfection of RNAs using a cationic lipid-based transfection reagent. Oligo (dT)-purified circRNA (circRNA-Oligo (dT) is represented in blue, commercial control circRNA (circRNA-Ctrl) in yellow, circRNA purified by commercial silica-based capture columns (circRNA-kit) is represented in black, and 5-methoxyuridine-modified mRNA (mRNA) in grey. (**B**) HeLa or (**C**) A549 cell lines were transfected with 100 ng/well of circRNA-Oligo (dT), circRNA-Ctrl, circRNA-kit, or mRNA. Luminescence was measured 24 h post-transfection. The x-axis in panel (**B**) is the same as in panel (**C**). (**D**) Scheme of the in vivo procedure to assess translation efficacy of the different RNAs encapsulated into LNPs. (**E**) In vivo luminescence levels monitored over 144 h (6 days) following intramuscular injection of LNPs encapsulating circRNA-Oligo (dT), circRNA-Ctrl, or mRNA in a mouse model. All LNPs were formulated using the commercial ionizable lipid SM-102, maintaining consistent lipid molar ratios across all samples. (**F**) Area under the curve (AUC) analysis of cumulative luciferase expression over the 6-day period represented in **E**. Results are represented as mean ± SD. Statistical significance was determined using one-way ANOVA with Tukey’s post hoc test (*: *p*-value < 0.05; ***: *p*-value < 0.001; *ns*: *p*-value > 0.05).

We next assessed the translational capacity of circRNA purified by Oligo (dT) affinity chromatography in vitro, comparing it to circRNA purified by HPLC (circRNA-Ctrl) as a benchmark [35]. Luminescence assays conducted 24 h post-transfection (Figure 3A) showed that circRNA-Oligo (dT) produced 14-fold higher protein levels compared to the commercial circRNA-Ctrl, and 9-fold compared to a circRNA purified by commercial silica-based capture columns (circRNA-kit) in HeLa cells. Additionally, circRNA-Oligo (dT) exhibited a 2-fold increase in protein production compared to conventional *N*1-methylpseudouridine-modified mRNA (Figure 3B). Similar trends were observed in HEK293T (Figure S3) and A549 cells (Figure 3C). Notably, the difference was most pronounced in A549 cells, where circRNA-Oligo (dT) generated 25-fold higher luminescence than circRNA-Ctrl purified using the HPLC method [35] and 9-fold compared to circRNA-kit. This is particularly significant given that A549 cells possess functional Toll-like receptors (TLRs), making them more sensitive to circRNA impurities than HEK293T cells [13]. Furthermore, these transfection results align with our dsRNA impurity data, which showed higher levels of impurities in circRNA-kit than in circRNA-Oligo (dT) (Figure 2).

Given that circRNA is intended for use in in vivo therapies, we next assessed the translational capacity of circRNA purified using our new method in a mouse model. We encapsulated circRNA-Oligo (dT), circRNA-Ctrl, and mRNA into conventional LNPs using ionizable lipid SM-102. Following intramuscular administration of RNA-LNPs, luminescence was monitored over 6 days. circRNA-Oligo (dT) showed higher luminescence values than circRNA-Ctrl from the time point at 4 h (Figure 3D,E) and also surpassed those of modified mRNA. Area under the curve (AUC) analysis over the 6-day period indicated that circRNA-Oligo (dT) produced a significantly higher cumulative amount of luciferase protein than circRNA-Ctrl (Figure 3F). Although circRNA-Oligo (dT) produced a similar cumulative amount of protein as mRNA, the persistence of the signal was markedly different. By day 3 (72 h), luminescence from circRNA-Oligo (dT) was 3.7 times higher than that of mRNA, indicating that circRNA-Oligo (dT) may be better suited for therapies requiring sustained protein expression over time.

### 2.4. CircRNA-Oligo (dT) Encapsulated with Novel Ionizable Lipids Shows Long-Lasting Protein Production In Vivo and Lyophilizability

**Figure 4:**
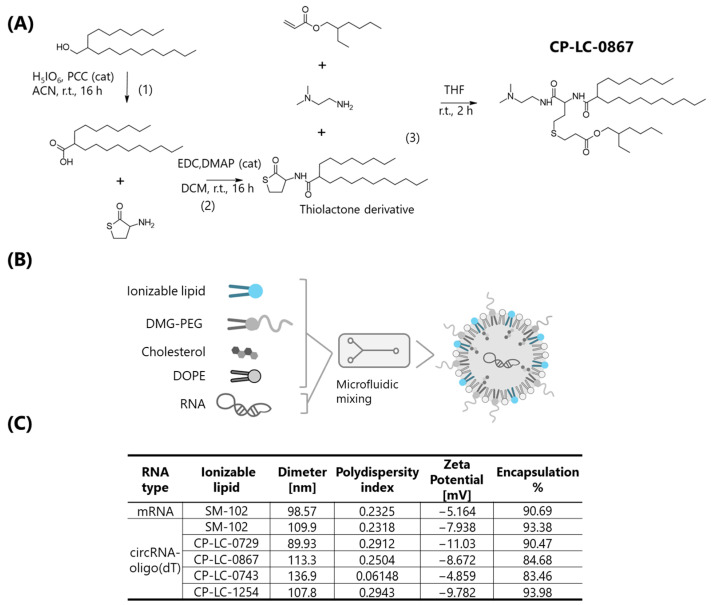
Synthesis of CP-LC-0867 and physicochemical characterization of circRNA-LNP formulations. (**A**) Schematic representation of the synthetic route to CP-LC-0867: (1) oxidation of the hydrophobic alcohol to a carboxylic acid, (2) amide bond formation between the thiolactone and the carboxylic acid, and (3) a one-pot, multicomponent reaction involving the thiolactone derivative, an acrylate, and a primary amine to yield CP-LC-0867. (**B**) Schematic representation of the encapsulation of circRNA or mRNA into lipid nanoparticles (LNPs) by microfluidic mixing. (**C**) Table summarizing the physical properties of LNP formulations containing circRNA or mRNA and the indicated ionizable lipid. The measured parameters include particle diameter (in nanometers), polydispersity index (PDI) for uniformity, zeta potential (in mV) for surface charge, and encapsulation efficiency (percentage of RNA encapsulated).

**Figure 5:**
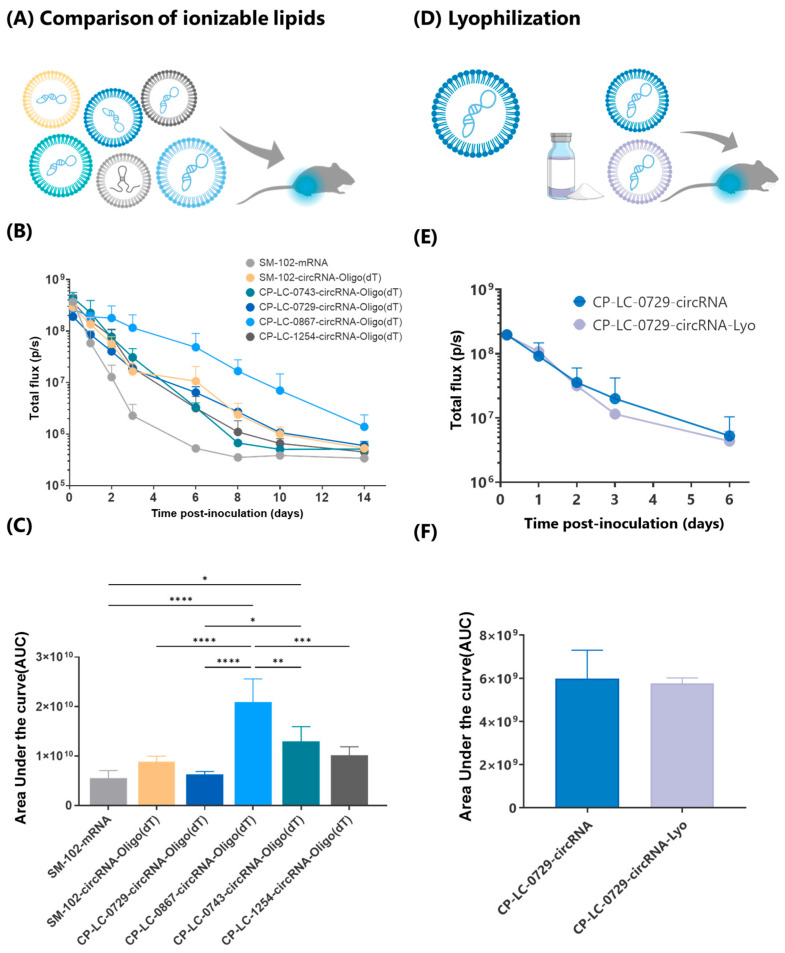
In vivo comparison of different ionizable lipids and lyophilization impact on circRNA-Oligo (dT) LNP. (**A**) Schematic of in vivo comparison of different ionizable lipids used to encapsulate circRNA-Oligo (dT) into LNPs. Control using mRNA as cargo is indicated in light grey and the different ionizable lipids in green, yellow, black, and light or dark blue. (**B**) In vivo luminescence monitoring over a 14-day period following intramuscular injection in mice of LNPs encapsulating circRNA-Oligo (dT) or mRNA. Ionizable lipid used for the LNP formulation is indicated. (**C**) Comparison of cumulative luciferase protein expression (AUC) over the entire monitoring period in panel (**B**). (**D**) Experimental workflow illustrating the lyophilization of LNPs encapsulating circRNA-Oligo (dT) using CP-LC-0729 as the ionizable lipid. Colors in panels C and D represent the different LNP formulations used and are uniformly applied in both panels for direct comparison. (**E**) Luminescence monitoring over a 6-day period following intramuscular injection in mice, comparing lyophilized and non-lyophilized LNP formulations. (**F**) Cumulative luciferase protein expression, calculated via the area under the curve (AUC) of panel (**E**). Results are represented as mean ± SD. Statistical significance was determined using one-way ANOVA with Tukey’s post hoc test (*: *p*-value < 0.05; **: *p*-value < 0.01; ***: *p*-value < 0.001; ****: *p*-value < 0.0001, absence of asterisks: *p*-value > 0.05).

To further develop an industrially scalable circRNA therapy, it is essential to expand the range of ionizable lipids available for LNP encapsulation. We have developed a broad family of ionizable lipids [27], originally evaluated for mRNA encapsulation and delivery, but with applicability for encapsulating any potential type of nucleic acid. In this study, we focused on evaluating the top three candidates from this family, CP-LC-0729, CP-LC-0743, and CP-LC-1254 (Figure S4), specifically for their efficiency in delivering circRNA-Oligo (dT) in vivo. In addition, we introduced a novel lipid, CP-LC-0867, synthesized using the same platform (Figure 4A), whose structure, although not yet published in the scientific literature, is described in a recent patent [28]. Each LNP formulation was physically characterized (Figure 4C), tested in vitro (Figure S5), and evaluated in a mouse model via intramuscular injection to assess its effectiveness in circRNA delivery and subsequent protein expression (Figure 5A).

Notably, while luminescence was initially planned to be monitored over a 6-day period post-injection, LNPs formulated with CP-LC-0867 exhibited sustained high levels of luciferase expression, prompting us to extend the monitoring period to 14 days. LNPs containing CP-LC-0867 consistently produced higher protein levels compared to those formulated with SM-102, while LNPs with CP-LC-0729 exhibited a similar expression profile to SM-102 (Figure 5B). CP-LC-0743 and CP-LC-1254 also showed similar expression profiles to SM-102, but with a more pronounced decrease in protein levels from 72 h onward. Interestingly, these in vivo results did not align with the in vitro data from HeLa, HEK293T, or A549 cells (Figure S5), where CP-LC-0867 performed similarly to the other lipids. This discrepancy may be due to the shorter time points used in cell-based luminescence assays compared to the extended monitoring in mice. However, it is more likely attributable to the well-documented differences between in vitro and in vivo transfection efficiencies of LNPs, as reported extensively in the literature [27].

When analyzing cumulative luciferase protein expression, as measured by the area under the curve (AUC), CP-LC-0867 generated significantly higher protein levels compared to all other conditions (Figure 5C). Although the AUC analysis showed similar total luciferase production between mRNA and certain circRNA formulations, this was largely due to mRNA producing higher protein levels at early time points, which skews the overall comparison. However, at an intermediate time point of 6 days, the sustained protein production profile became more evident, with CP-LC-0867 exhibiting a 92-fold increase in protein expression and the remaining circRNA formulations showing at least a 6-fold increase compared to mRNA-LNP formulations. These results highlight the long-term stability and effectiveness of circRNA-LNPs, particularly those formulated with CP-LC-0867.

We then assessed the lyophilization capacity of LNP-circRNA-Oligo (dT) to extend its storage life and eliminate the need for ultra-low-temperature storage and transport, which represents a significant hurdle to the broader adoption of circRNA therapies. For this study, we selected CP-LC-0729 as the ionizable lipid, as it is one of the most thoroughly characterized lipids among the new ionizable lipids we have tested. Using a previously established lyophilization method [29], we lyophilized LNPs encapsulating circRNA-Oligo (dT) with CP-LC-0729 and evaluated their physical characteristics (Figure S6) and functionality in mice, comparing them with non-lyophilized LNPs (Figure 5D). Lyophilization was also tested with CP-LC-0867, which showed similarly satisfactory results (Figure S7). After intramuscular administration and monitoring luminescence over a 6-day period, we observed no significant differences in the protein production profile (Figure 5E) or cumulative protein expression (Figure 5F) between lyophilized and non-lyophilized LNPs.

These results indicate that lyophilization does not impair the functionality of LNP-circRNA-Oligo (dT) using CP-LC-0729 or CP-LC-0867 as the ionizable lipid, making this new platform a viable option for extending storage life without the need for ultra-low-temperature conditions.

## 3. Discussion

Circular RNA has gained significant attention in therapeutic applications due to its exceptional stability and resistance to exonucleases. However, achieving efficient and scalable purification remains a considerable challenge. Traditional methods, such as PAGE electrophoresis and RNase R digestion, although effective, have significant limitations in terms of throughput, scalability, and the ability to achieve high purity [36]. PAGE, commonly employed for RNA separation based on size, conformation, and total molecular charge, provides high resolution but is labor-intensive and prone to contamination, which significantly reduces its practicality for large-scale operations [37]. Likewise, RNase R digestion, which selectively degrades linear RNA while preserving circRNA, often leaves structured linear contaminants and proves cost-prohibitive for large-scale purification. Consequently, while these methods are suitable for initial characterization, they fall short in terms of scalability and widespread industrial application [38].

High-performance liquid chromatography has also been explored for its potential to remove impurities such as dsRNA [9]. However, the HPLC technique reported for circRNA suffer from low recovery yields, as only a small fraction of the total RNA is collected, thus making the method challenging to scale for industrial use [9]. To overcome this scale restrictions, we have developed an innovative affinity chromatography method that for the first time employs Oligo (dT) columns to purify circRNA for therapy purposes. Additionally, we have eliminated the need for RNase R digestion, which provides a cost-effective, scalable alternative to HPLC.

In relation to Mg^2+^ concentration during the IVT process, previous reports largely rely on commercial kits where Mg^2+^ concentrations are undisclosed, making it impossible to modify or optimize [9,11,13]. In this study, we demonstrated that by adjusting the Mg^2+^ concentration, we can significantly increase the proportion of circRNA produced, providing an important optimization strategy for circRNA synthesis.

In terms of in vivo delivery of circRNA, only a limited number of studies have reported successful strategies. Some approaches have employed cationic polymers such as in vivo-jetPEI [11] or O6-stat-N6 CARTs, [3,6] while others have focused on LNPs using ionizable lipids like cKK-E12 [11], MC3 [2,39], SM-102, and ALC-0315 [38]. Nonetheless, several studies that utilize LNPs do not specify the ionizable lipid used [25]. Here, we report the best-performing ionizable lipid described to date, which demonstrated a remarkable 92-fold increase in in vivo protein expression at 6 days post-injection compared to SM-102. Moreover, while previous studies typically employed 5 to 20 μg of RNA for in vivo delivery [2,3,4,26,39], we achieved robust protein expression with just 1 μg of circRNA. This demonstrates the exceptional efficiency of our novel lipid formulations and their potential to significantly reduce the required therapeutic circRNA dosage.

A critical factor in evaluating circRNA for clinical applications is the duration of gene expression, as circRNA is designed to provide more sustained and stable expression compared to mRNA. To our knowledge, the longest previously reported in vivo signal duration is 42 h using LNPs [13] and 168 h (7 days) using O6-stat-N6 CARTs [6]. In this study, we report an extended signal duration of 336 h (14 days), doubling the 7-day expression window reported in the literature [6]. This prolonged expression suggests that the combination of Oligo (dT) purification and novel ionizable lipids provides superior performance over existing methods, making this approach particularly promising for long-term therapeutic applications.

Among the ionizable lipids evaluated in this study, CP-LC-0867 and CP-LC-0729 demonstrated not only superior in vivo efficacy but also notable advantages in terms of synthetic accessibility and scalability. The previously described synthesis of CP-LC-0729 involves just two steps, with the final step carried out as a straightforward one-pot reaction [27]. Similarly, CP-LC-0867, although requiring three synthetic steps, is produced under mild conditions without the need for stringent parameters [28]. In contrast, clinically established ionizable lipids such as SM-102 [40] and ALC-0315 [41] require four and three synthetic steps, respectively, and typically involve more demanding conditions, including high temperatures, reactions more prone to byproduct formation, and strictly anhydrous environments. These features highlight the practical advantages of CP-LC-0867 and CP-LC-0729 for large-scale and cost-effective manufacturing.

Lyophilization has been extensively studied to enhance the stability of mRNA vaccines [29], but its application to circRNA-LNPs remains largely unexplored, with only one prior study reported to date [26]. However, this study does not specify the ionizable lipid used and focuses on mannose-modified PEG for circRNA-LNP delivery to lymphocytes, without evaluating the impact of lyophilization on LNPs containing conventional, unmodified PEG. To our knowledge, our study provides the first demonstration of successful lyophilization of circRNA-LNPs using novel ionizable lipids [42] and standard PEGylated formulations. By evaluating both the physical integrity and functional preservation of the LNPs post-lyophilization, we offer a more comprehensive and translationally relevant analysis of this strategy’s potential to support broader storage and distribution of circRNA therapeutics.

Despite the promising results, our study has some limitations that warrant further investigation. First, while we have demonstrated enhanced circRNA yield and prolonged in vivo expression using Oligo (dT) purification and novel ionizable lipids, the evaluation was based on intramuscular injection. This route of administration is widely used for vaccines and other therapeutic applications, providing valuable insights into the platform’s effectiveness. However, it may not fully capture how the system would perform with alternative delivery routes, such as intravenous or subcutaneous injections. Exploring these routes in future studies will offer a broader understanding of the platform’s therapeutic potential and expand its applicability across various clinical scenarios. Additionally, we successfully lyophilized circRNA-LNPs, preserving their functionality, and developed an optimized and streamlined process for generating high-quality circRNA. Together, these advancements mark a qualitative breakthrough, setting the stage for more efficient and scalable circRNA therapeutic development. Looking ahead, we aim to explore the long-term stability of lyophilized circRNA-LNPs, building on our previous success with mRNA-LNPs stored for over a year at 4 °C [29], which included the use of CP-LC-0867 and other top-performing lipids. In addition, we will delve deeper into our extensive library of over 1500 ionizable lipids to identify even more effective candidates for circRNA delivery [42]. Furthermore, a thorough evaluation of the safety profile of our novel ionizable lipids will be essential in future studies to determine how they compare with clinically established standards.

In summary, our study provides an important step forward in the scalable production and in vivo delivery of circular RNA (circRNA), utilizing optimized purification strategies and novel ionizable lipids. We demonstrated that the use of Oligo (dT) affinity chromatography enhances the yield and purity of circRNA, allowing for efficient downstream applications without the need for additional enzymatic treatments. Furthermore, we successfully encapsulated circRNA into LNPs formulated with promising ionizable lipids, including CP-LC-0867 and CP-LC-0729, which resulted in superior protein expression profiles compared to conventional lipids such as SM-102. Additionally, our exploration of lyophilization revealed that lyophilized circRNA-LNP formulations retain their functionality, featuring their potential for long-term storage and global distribution. These results demonstrate the substantial potential of this platform to advance the clinical translation of circRNA therapies by enhancing production efficiency, stability, and delivery, which are critical factors for achieving reliable and scalable therapeutic outcomes.

## 4. Materials and Methods

### 4.1. Template Plasmid Construction for circRNA

The DNA template for circRNA synthesis was constructed by cloning the Anabaena 3.0 ribozyme, the internal ribosome entry site IRES-CVB3, both as previously described [9], and a firefly luciferase coding sequence optimized for human expression into a pUC-based plasmid under the control of a T7 promoter. Gene synthesis, cloning, and plasmid preparations were outsourced to Genscript (Piscataway, NJ, USA).

Subsequently, the plasmid was linearized with the BspQI restriction enzyme (Hongene, Shanghai, China) at 50 °C for 2 h. The linearized plasmid was then purified using the Wizard^®^ SV gel and PCR clean-up system kit (Promega, Madison, WI, USA) following the manufacturer’s instructions.

### 4.2. Synthesis of RNA and Enrichment of circRNA

Modified linear mRNA and unmodified circRNA were synthesized by in vitro transcription (IVT) using T7 RNA polymerase (Hongene). The transcription reaction was set up with 1 µg of linearized plasmid DNA, 100 U of T7 RNA polymerase, 5 mM rNTPs, and 1× transcription buffer in a total volume of 20 µL. IVT reaction was incubated at 37 °C for 3 h. Following the IVT reaction, the DNA template was digested with 4 U of DNase I (Hongene) at 37 °C for 15 min.

For circRNA synthesis optimization, we systematically varied the IVT incubation times (1, 2, 3, 5, 7, or 16 h) and adjusted the concentration of Mg^2+^ in the transcription buffer to 5, 10, 16.5, or 20 mM.

To enrich the circRNA, the IVT product was denatured at 70° C for 5 min and then immediately placed on ice for 3 min. Subsequently, GTP was added to a final concentration of 2 mM, and the mixture was incubated at 55 °C for 15 min. The reaction was then quenched by the addition of 5 mM EDTA at pH 8 and storage for its subsequent purification.

The circRNA used in this study has an approximate molecular weight of 829.1 kDa, corresponding to 2573 nucleotides. The control circular RNA (circRNA-Ctrl) used in this study was obtained from GenScript (SC8888), where it was purified via high-performance liquid chromatography (HPLC) [35]. This control circRNA contains the same elements as the experimental circRNA constructs produced in our laboratory, ensuring consistency in evaluating the effects of various conditions on circRNA functionality and stability [35].

### 4.3. Purification and Identification of circRNA

Resulting RNA transcripts were purified by affinity chromatography using POROS Oligo (dT) 25 (ThermoFisher, Bedford, MA, USA). The columns were equilibrated with 5 column volumes of binding buffer (50 mM sodium phosphate, 0.5 M NaCl, 5 mM EDTA, pH = 7.0) before use. The RNA solution was applied to the equilibrated Oligo dT column. The column was washed with 10 column volumes of wash buffer (50 mM sodium phosphate, 5 mM EDTA, pH = 7.0) and was eluted by applying 2 column volumes of double-deionized water. The eluate was collected and immediately placed on ice. A representative chromatogram is presented in Figure S2, illustrating the separation profile achieved during the purification process.

Subsequently, a polishing treatment of the RNAs was performed to enrich the circular isoform relative to the other species. Resulting RNA fractions were visualized on a 4% polyacrylamide gel (PAGE) containing 8 M urea and analyzed using the FIJI software (version Image J2).

The circRNA-Kit was purified using the Monarch^®^ Spin RNA Cleanup Kit (NEB, Ipswich, MA, USA) (50 μg), following the manufacturer’s instructions for optimal RNA recovery.

The quality and size of the resulting RNA samples were evaluated using a Bioanalyzer Agilent 2100 (Agilent Technologies, Waldbronn, Germany), with the Agilent RNA 6000 Nano kit. The resulting data were processed and evaluated with 2100 Expert Software (Version: B.02.08.SI648 (SR2)) RNA quality was further evaluated using high-performance liquid chromatography (HPLC). Samples were processed through a CIMac™ SDVB 0.1 mL (BiA Separations, Ajdouscina, Slovenia) analytical reverse-phase column (2 µm channels, Sartorius) connected to an HPLC WATERS e2695. CIMac SDVB column was equilibrated at 65 °C in mobile phase A (50 mM TEAA and 7.5% ACN, pH 7.0). Approximately 0.4 µg RNA was injected on the column. After an initial equilibration step (1 min at 100% MPA), a linear gradient was performed, 0–100% B (50 mM TEAA in 18% CAN pH = 7.0) in 27 min at a flow rate 1 mL min^–1^. The column was eluted with 100% MPC (50 mM TEAA in 75% CAN pH = 8.2) for 1 min before re-equilibration in 100% MPA.

### 4.4. Determination of dsRNA Content

The circRNAs were spotted onto positively charged nylon membranes (Nytran SC, Cytiva, München, Germany). The membranes were then blocked with 5% (*w*/*v*) non-fat dried milk in TBS-T buffer (20 mM Tris–HCl, 150 mM NaCl, 0.1% [*v*/*v*] Tween-20, pH 7.4), and incubated with dsRNA-specific mAb J2 (Jena Bioscience, Jena, Germany) at 4 °C overnight. Membranes were washed with TBS-T buffer and incubated with HRP-conjugated goat anti-mouse IgG (Abcam, Cambridge, UK). Following additional wash with TBS-T, the membranes were treated with ECL Plus Western blot detection reagent (Amersham, Chicago, IL, USA). Images were captured using an iBright 750 digital imaging system.

### 4.5. Synthesis and Characterization of Ionizable Lipids

The ionizable lipids CP-LC-0743, CP-LC-0729, and CP-LC-1254 were synthesized following established protocols based on the Sequential Thiolactone Amine Acrylate Reaction [27,42]. In brief, thiolactone derivatives (0.15 mmoL, 1 equiv.) and acrylate (0.15 mmoL, 1 equiv.) were dissolved in 300 μL of tetrahydrofuran (THF) at room temperature (RT), followed by the addition of the amine (0.15 mmoL, 1 equiv). After two hours, the THF was removed under vacuum. Notably, CP-LC-0743 was previously referred to as A4B2C1, and CP-LC-1254 was previously designated as CP-LC-0729-04 [27].

The synthesis of CP-LC-0867 was carried out as follows: 2-Octyldecanoic acid: 2-octyl-1-dodecanol (1077 mg, 3.5 mmoL) and periodic acid (4144 mg, 18 mmoL) were dissolved in acetonitrile (28 mL). Then, pyridinium chlorochromate (23 mg, 0.030 mmoL) was added to the previous solution. The reaction mixture was stirred 16 h at room temperature. Then, the solvent was evaporated under reduced pressure. Subsequently, the crude was dissolved in AcOEt and first washed twice with water and finally with brine. The organic phase was dried with anhydrous MgSO_4_, filtered, and evaporated under reduced pressure. The residue was used in the next step without any further purification.

Thiolactone derivative (2-octyl-N-(2-oxotetrahydrothiophen-3-yl) dodecanamide): DL-homocysteine thiolactone hydrochloride (1.8 mmoL) was dissolved in 5 mL of anhydrous dichloromethane at room temperature. Then, triethylamine (1.8 mmoL) was added followed by EDC hydrochloride (1.3 mmoL), 4-(dimethylamino) pyridine (0.20 mmoL), and 2-octyldecanoic acid (1.0 mmoL). The reaction mixture was stirred at room temperature overnight under an argon atmosphere. The organic layer was dried with anhydrous MgSO4, filtered, and evaporated under reduced pressure. The resulting residue was purified by flash chromatography (gradient of hexane/ethyl acetate: 100/0 to 0/100) to afford as a pure product.

MS-QDa: Theorical [M + H]^+^ = 412.32, experimental [M + H]^+^ = 412.50.

Thiolactone derivative [27] (0.15 mmoL, 1 equiv.) and 2-ethylhexyl acrylate (0.15 mmoL, 1 equiv.) were dissolved in 300 μL of THF at RT, followed by the addition of N,N-dimethylethylenediamine (0.15 mmoL, 1 equiv.). After two hours, the THF was removed under vacuum, and CP-LC-0867 was purified using a CombiFlash NextGen 300+ with gradient elution, starting from 100% dichloromethane to 50% of an 80/20/1 mixture of DCM/MeOH/NH_4_OH (aq).

All synthesized lipids were analyzed by high-performance liquid chromatography (HPLC) equipped with a charged aerosol detector (CAD) and characterized by mass spectrometry (ISQ ThermoFisher, Waltham, MA, USA). The structures of the synthesized lipids are available in Figure S4. ^1^H-RMN data are shown in Figures S8 and S9 and summarized below:

- CP-LC-0867: MS-QDa: theoretical [M + H]^+^ = 684.57, experimental [M + H]^+^ = 684.58; ^1^H NMR (500 MHz, CDCl3) δ 6.63 (t, *J* = 5.1 Hz, 1H), 6.25 (d, *J* = 7.9 Hz, 1H), 4.59 (dt, *J*= 7.2 Hz *J* = 7.2 Hz, 1H), 4.07–3.96 (m, 2H), 3.32 (dt, *J* = 6.4, 5.5 Hz, 2H), 2.80 (t, *J* = 7.3 Hz, 2H), 2.67–2.49 (m, 4H), 2.40 (t, *J* = 6.1 Hz, 2H), 2.22 (s, 6H), 2.11–1.99 (m, 2H), 1.94 (m, 1H), 1.57 (m, 3H), 1.45–1.32 (m, 4H), 1.25 (m, 34H), 0.88 (m, 12H).
- CP-LC-0743: MS-QDa: theoretical [M + H]^+^ = 766.25, experimental [M + H]^+^ = 766.81; ^1^H NMR (400 MHz, CDCl3) δ 6.64 (t, *J* = 4.8 Hz, 1H), 6.26 (d, *J* = 8.0 Hz, 1H), 5.39–5.29 (m, 2H), 4.59 (dt, *J* = 7.2 Hz *J* = 7.2 Hz, 1H), 4.08 (t, *J* = 6.8 Hz, 2H), 3.32 (dt, *J* = 5.9 Hz *J* = 5.9 Hz, 2H), 2.79 (t, *J* = 7.2 Hz, 2H), 2.67–2.48 (m, 4H), 2.40 (t, *J* = 6.1 Hz, 2H), 2.22 (s, 6H), 2.11–1.88 (m, 7H), 1.59 (m, 4H), 1.46–1.15 (m, 44H), 0.87 (m, 9H).
- CP-LC-0729: MS-QDa: theoretical [M + H]^+^ = 740.63, experimental [M + H]^+^ = 740.85; ^1^H NMR (400 MHz, CDCl3) δ: 6.76 (m, 1H); 6.37 (d, *J* = 7.9 Hz, 1H); 4.60 (dt, *J* = 7.1 Hz *J* = 7.1 Hz, 1H); 3.98 (d, *J* = 5.8 Hz, 2H); 3.36 (dt, *J* = 5.7 Hz *J* = 5.7 Hz, 2H); 2.78 (t, *J* = 6.8 Hz, 2H); 2.59 (m, 4H); 2.48 (t, *J* = 5.9 Hz, 2H); 2.28 (s, 6H); 2.06 (m, 2H); 1.96 (m, 1H); 1.58 (m, 3H); 1.41 (m, 2H); 1.25 (m, 44H); 0.87 (m, 12H).
- CP-LC-1254: MS-QDa: theoretical [M + H]^+^ = 755.63, experimental [M + H]^+^ = 755.83; ^1^H NMR (400 MHz, CDCl3) δ: 6.79 (m, 1H); 4.05 (d, *J* = 5.8 Hz, 2H); 4.01 (d, *J* = 5.8 Hz, 2H); 3.45 (t, *J* = 7.1 Hz, 1H); 3.36 (m, 2H); 2.79 (t, *J* = 7.6 Hz, 2H); 2.69–2.49 (m, 4H); 2.44 (t, *J* = 6.0 Hz, 2H); 2.26 (s, 6H); 2.21 (m, 2H); 1.65 (m, 2H); 1.29 (m, 48H); 0.90 (m, 12H).

### 4.6. Lipid Nanoparticle (LNP) Formulation and Characterization

The synthesis of LPNs was performed using a microfluidic method. An ethanolic lipid solution containing the synthetized CP-LC ionizable lipids or the commercial SM-102 (BocSCI, Shirley, NY, USA), DOPE (Corden Pharma, Plankstadt, Germany), cholesterol (Merk, Rahway, NJ, USA), and DMG-PEG2000 (Cayman, Ann Arbor, MI, USA) at a molar ratio of 50:10:38.5:1.5 was prepared. This lipid mixture was then combined with an aqueous phase containing mRNA or circRNA diluted in 10 mM citrate buffer (pH 4), achieving an ionizable lipid/RNA weight ratio of 10:1. LNPs were formulated using an INano^TM^ microfluidic device set to a total flow rate of 12 mL/min with an aqueous-to-ethanol volume ratio of 3:1. After LNP formation, ethanol was removed by dialysis (Pur-A-Lyzer™ Midi Dialysis Kit, Sigma-Aldrich, St. Louis, MO, USA).

For LNP characterization the average size, polydispersity (PDI), and zeta potential of LNPs were determined using a Malvern Zetasizer Advance Lab Blue Label (Malvern Instruments Ltd., Malvern, UK). Concentration of encapsulated RNA (circRNA or mRNA) was then quantified using the Quant-IT^®^ Ribogreen assay (Invitrogen, Waltham, MA, USA) following the manufacturer’s instructions, and LNP encapsulation efficiency were verified by agarose gel electrophoresis.

The final LNP formulation was adjusted to an RNA (circRNA or mRNA) concentration of 100 μg/mL and was subsequently lyophilized and/or stored at 4 °C for subsequent in vivo and in vitro analysis.

### 4.7. LNP Lyophilization

Lyophilization was performed following previously reported protocols, [29] using a Virtis Genesis Pilot Freeze Dryer (SP, Warminster, PA, USA). In brief, LNPs were lyophilized with 20% maltose as a lyoprotectant in TRIS buffer. The process included three stages: (1) freezing at −50 °C, (2) primary drying at −10 °C under 180 mTorr pressure, and (3) secondary drying at 40 °C, gradually reducing the pressure to 120 mTorr and finally to 50 mTorr. Upon completion, the vials were backfilled with pure nitrogen to maintain an inert atmosphere, sealed, and stored at various temperatures for stability assessments. For reconstitution, 300 μL of RNase-free water was added to each vial, and the contents were gently swirled until a homogenous suspension was obtained.

### 4.8. Cell Culture and circRNA Transfection

The HeLa (DSMZ GmbH, Braunschweig, Germany), HEK293T (ATCC; American Type Culture Collection, Manassas, VA, USA).) and A549 (NIBSC; (National Institute for Biological Standards and Control, South Mimms, Hertfordshire, UK).) cell lines were maintained in DMEM with high glucose (Merk, Darmstadt, Germany), supplemented with 10% Fetal Bovine Serum (Sigma, Darmstadt, Germany), 1% Penicillin-Streptomycin Solution (GibcoTM, Thermo Fisher Scientific, Waltham, MA, USA) and 2 mM Glutamax (Thermo Fisher Scientific, Waltham, MA, USA). All cell lines were grown in 175 cm^2^ flasks.

The day before transfection, cells were detached from culture flasks using trypsinization and subsequently seeded into 96-well plates at a density of 1 × 10^4^ cells per well. Immediately before the transfections, the culture media in each well was replaced with 90 μL of fresh media. For RNA (circRNA or mRNA) transfection using a cationic lipid-based transfection reagent a mixture containing RNA and Lipofectamine MessengerMAX™ (Invitrogen, Thermo Fisher Scientific, Waltham, MA, USA) was pre-incubated in OptiMEM (GibcoTM, Thermo Fisher Scientific, Waltham, MA, USA) media according to the manufacturer’s instructions. This RNA-lipofectamine complex was then added to the designated wells in triplicate, achieving a final RNA concentration of 100 ng/well.

For RNA-LNP transfection a suspension of RNA-containing LNPs was added to the designated wells in triplicate to achieve a final RNA concentration of 200,100 and 50 ng/well. The cells were then incubated for 24 h at 37 °C in a 5% CO_2_ atmosphere to allow translation of the delivered RNA into luciferase protein.

### 4.9. Luciferase Activity Quantification In Vitro

At 24 or 48 h post-transfection, the cells were lysed by adding 100 μL of PBS containing 0.1% Triton X-100 to each well and incubating for 10 min at room temperature. This lysis step allows the release of luciferase protein translated from the delivered circRNA or mRNA. Following lysis, 98 μL of the cell lysate was transferred to an opaque 96-well white plate. To each well, 100 μL of a buffered d-Luciferin solution (GoldBio, Olivette, MO, USA) containing 100 mM Tris-HCl (pH 7.8), 5 mM MgCl_2_, 250 μM CoA, and 150 μM ATP was added, resulting in a final d-Luciferin concentration of 150 μg/mL. Luminescence was measured after a 5 min incubation at room temperature using a FLUOstar Omega (version 5.70) plate reader (BMG LABTECH, Ortenberg, Germany). Cells that had not been treated with any mRNA were used as a negative control to assess background luminescence.

### 4.10. Administration of LNP-circRNAs in Mice

Female BALB/c mice (Janvier), aged 8–10 weeks and weighing 18–23 g, were acclimatized to the experimental facilities for 3–7 days upon arrival. The mice were housed under controlled conditions, maintaining a room temperature of 20–24 °C, humidity levels between 50 and 70%, and a light intensity of 60 lux with a 12 h light–dark cycle.

For in vivo measurement of Firefly Luciferase activity, LNPs containing 1 μg of the specified RNA in a final volume of 30 μL. were administered via intramuscular injection. At the designated post-injection times, mice were anesthetized with 4% isoflurane administered via a vaporizer for induction, and anesthesia was maintained at 1.5% isoflurane. Following anesthesia, D-luciferin (Quimigen, Madrid, Spain) was administered intraperitoneally at a dose of 150 mg/kg. Ten minutes after D-luciferin administration, luminescence images were captured using the IVIS Lumina XRMS Imaging System (Living Image software version 4.8.2).

## Data Availability

The original contributions presented in this study are included in the article/supplementary material. Further inquiries can be directed to the corresponding authors.

## Supplementary Materials

The following supporting information can be downloaded at: https://www.mdpi.com/article/10.3390/ijms26115138/s1.
