# Supplementary material for: A Complete Approach for circRNA Therapeutics from Purification to Lyophilized Delivery Using Novel Ionizable Lipids
Source: Int J Mol Sci. 2025 May 27;26(11):5138. doi: 10.3390/ijms26115138 (PMC12154124; doi:10.3390/ijms26115138)
Supplement: Supplementary file 1 [file ijms-26-05138-s001.zip › ijms-3615177-supplementary.pdf]

(A)

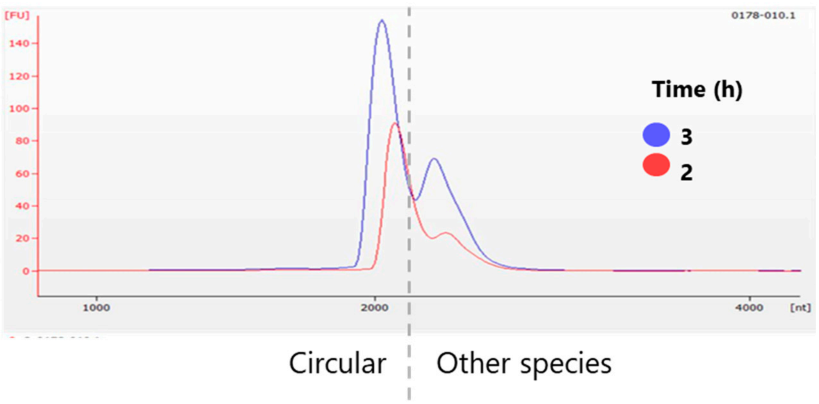

(B)

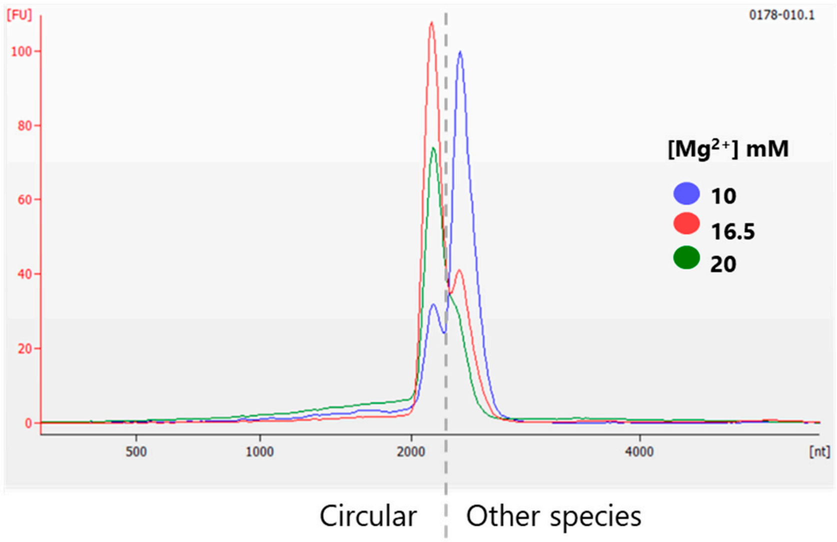

Figure S1. Capillary electrophoresis histograms **(A)** at various incubation times during the in vitro transcription (IVT) reaction optimization and **(B)** with various Mg^{2+} concentrations. The left peak corresponds to the circular RNA isoforms, while the right peak corresponds to the remaining RNA species.

(A)

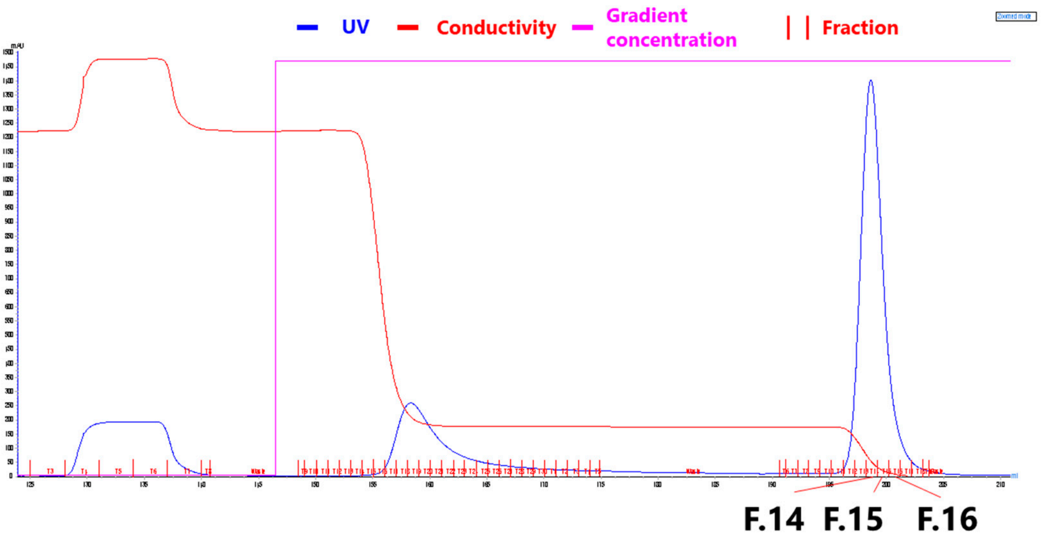

(B)

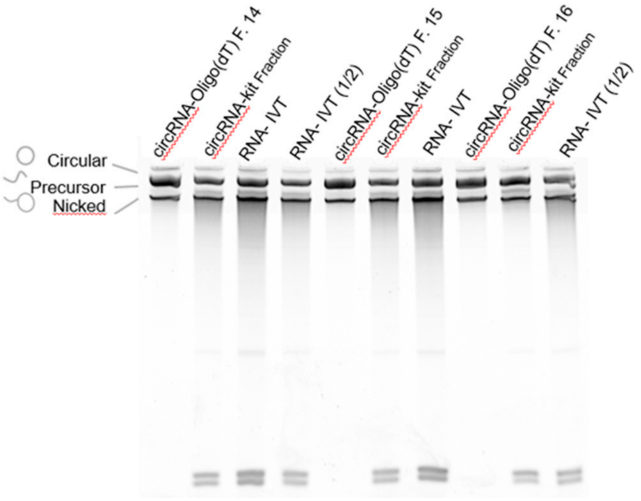

Figure S2. (A) Full chromatographic profile obtained during the purification of circular RNA using an ÄKTA system equipped with an Oligo(dT) affinity column. The x-axis represents the elution volume (mL), and the y-axis corresponds to absorbance at 280 nm (mAU). The major peak observed at 199 ml corresponds to the elution of purified circRNA. Fractions 14, 15 and 16, corresponding to this peak were collected and subsequently analyzed for integrity and purity by gel electrophoresis. **(B)** Comparison of circRNA purity in fractions obtained under different purification conditions. Denaturing PAGE (4% acrylamide, 8 M urea) showing RNA samples obtained from various purification steps.

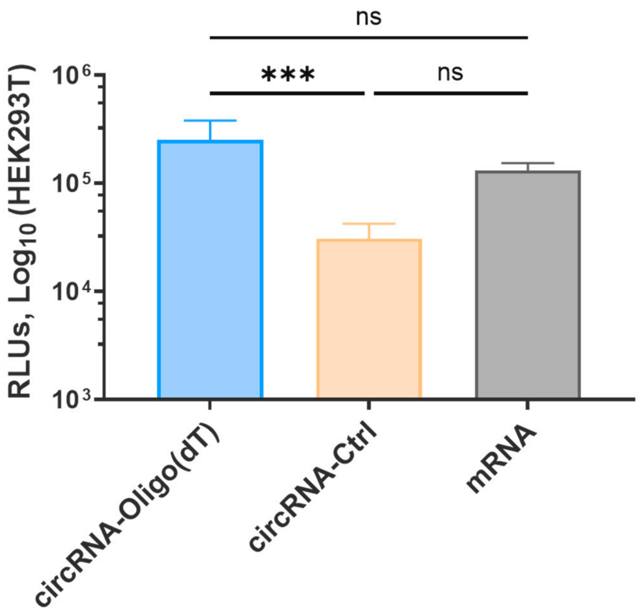

Figure S3. Luciferase production measured in Relative Luminescence Units (RLU) in HEL293T cell line transfected with 100 ng/well of circRNA-Oligo(dT), circRNA-Ctrl or mRNA. Luminescence was measured 24 hours post-transfection. Results are represented as mean ± SD. Statistical significance was determined using one-way ANOVA with Tukey's post-hoc test (***: *P*-value <0.001; *ns*: *P*-value >0.05).

CP-LC-0743

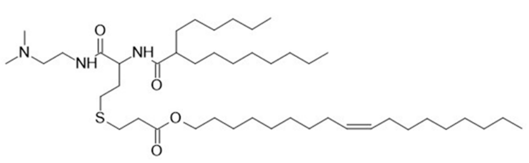

CP-LC-0729

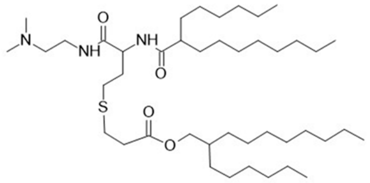

CP-LC-1254

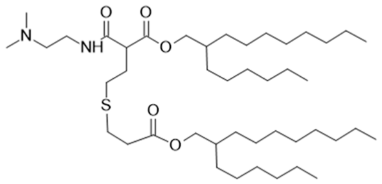

CP-LC-0867

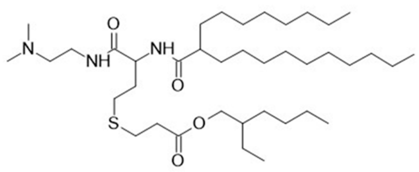

Figure S4. Structure of CP-LC ionizable lipids used in the study.

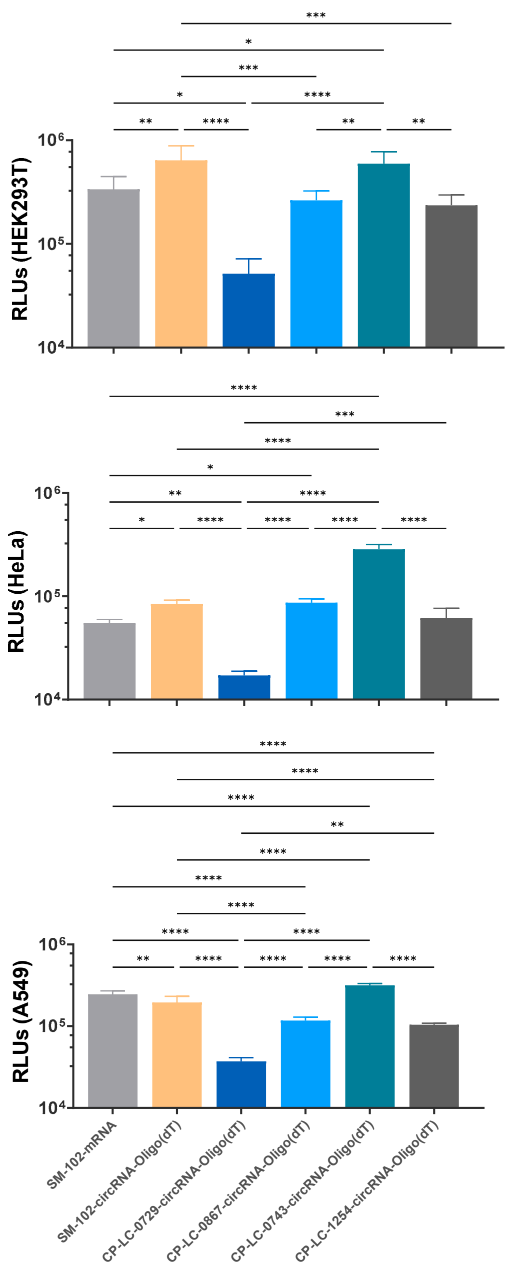

Figure S5. Luciferase production measured in Relative Luminescence Units (RLU) in HEK293T, HeLa and A549 cell lines transfected with 100 ng/well of LNPs encapsulating circRNA-Oligo(dT) or mRNA with the indicated ionizable lipids. Luminescence was measured 24 hours post-transfection. Results are represented as mean ± SD. Statistical significance was determined using one-way ANOVA with Tukey's post-hoc test (*: P-value <0.05**; P-value <0.01; ***: P-value <0.001; ****: P-value <0.0001 ns: P-value >0.05).

RNA type	Formulation	Ionizable lipid	Dimeter [nm]	Polydispersity index	Zeta Potential [mV]	Encapsulation %
circRNA-oligo(dT)	Liquid	CP-LC-0729	92,84	0,2342	-14,49	96,10
circRNA-oligo(dT)	Lyophilized	CP-LC-0729	101,9	0,1552	-11,8	86,20

Figure S6. Table summarizing the physical properties of LNP formulations containing circRNA-Oligo(dT) and CP-LC-0729 ionizable lipid either lyophilized or not. The measured parameters include particle diameter (in nanometers), polydispersity index (PDI) for uniformity, zeta potential (in mV) for surface charge, and encapsulation efficiency (percentage of RNA encapsulated).

(A)

RNA type	Formulation	Ionizable lipid	Dimeter [nm]	Polydispersity index	Zeta Potential [mV]	Encapsulation %
circRNA-oligo(dT)	Liquid	CP-LC-0867	126.1	0.1107	-10.51	88.44
circRNA-oligo(dT)	Lyophilized	CP-LC-0867	144.6	0.1288	-16.24	83.18
mRNA	Liquid	SM-102	99.97	0.1728	-7.669	99.38

(B)

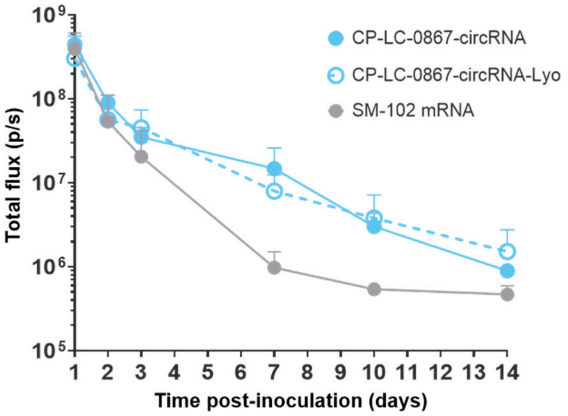

Figure S7. (A) Table summarizing the physical properties of LNP formulations containing circRNA-Oligo(dT) and CP-LC-0867 ionizable lipid either lyophilized or not and containing mRNA and SM-102. The measured parameters include particle diameter (in nanometers), polydispersity index (PDI) for uniformity, zeta potential (in mV) for surface charge, and encapsulation efficiency (percentage of RNA encapsulated). **(B)** Luminescence monitoring over a 14-day period following intramuscular injection in mice, comparing lyophilized and non-lyophilized LNP formulations with CS-LC-0867 ionizable lipid.

(A)

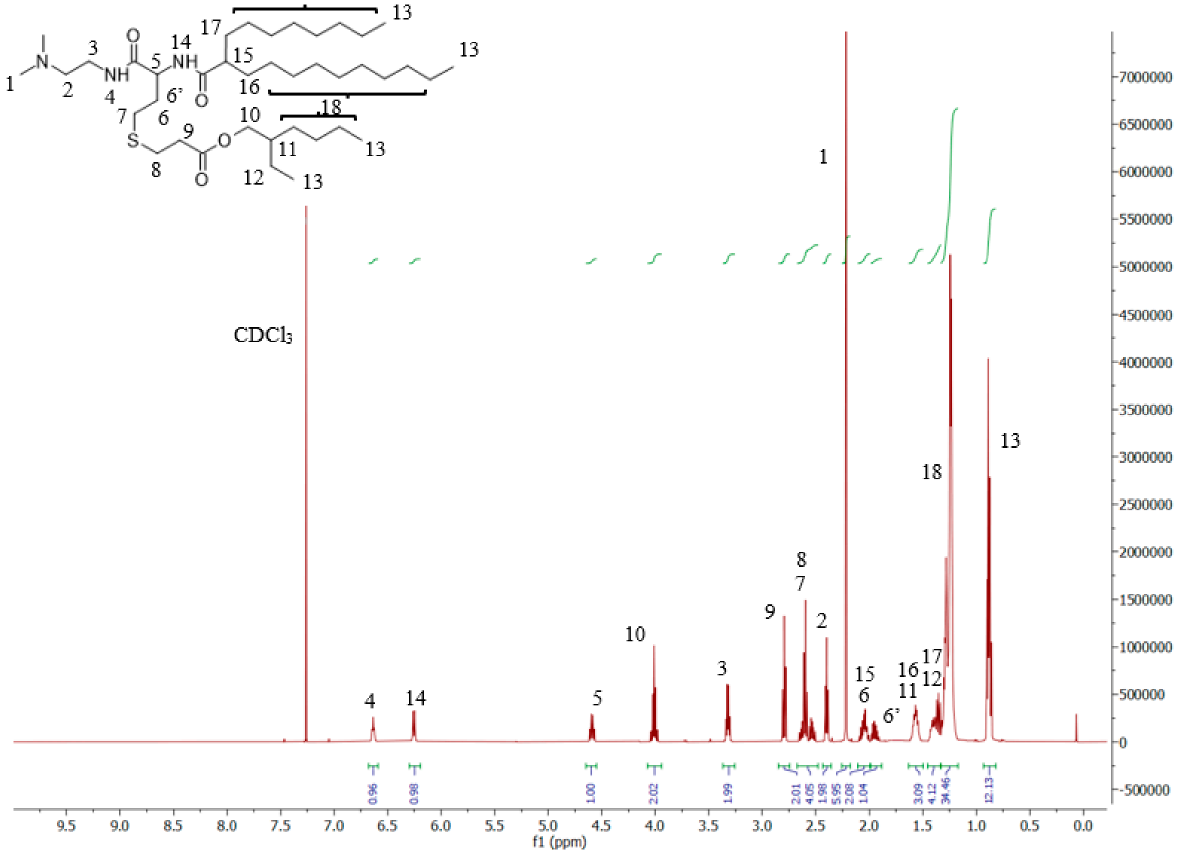

(B)

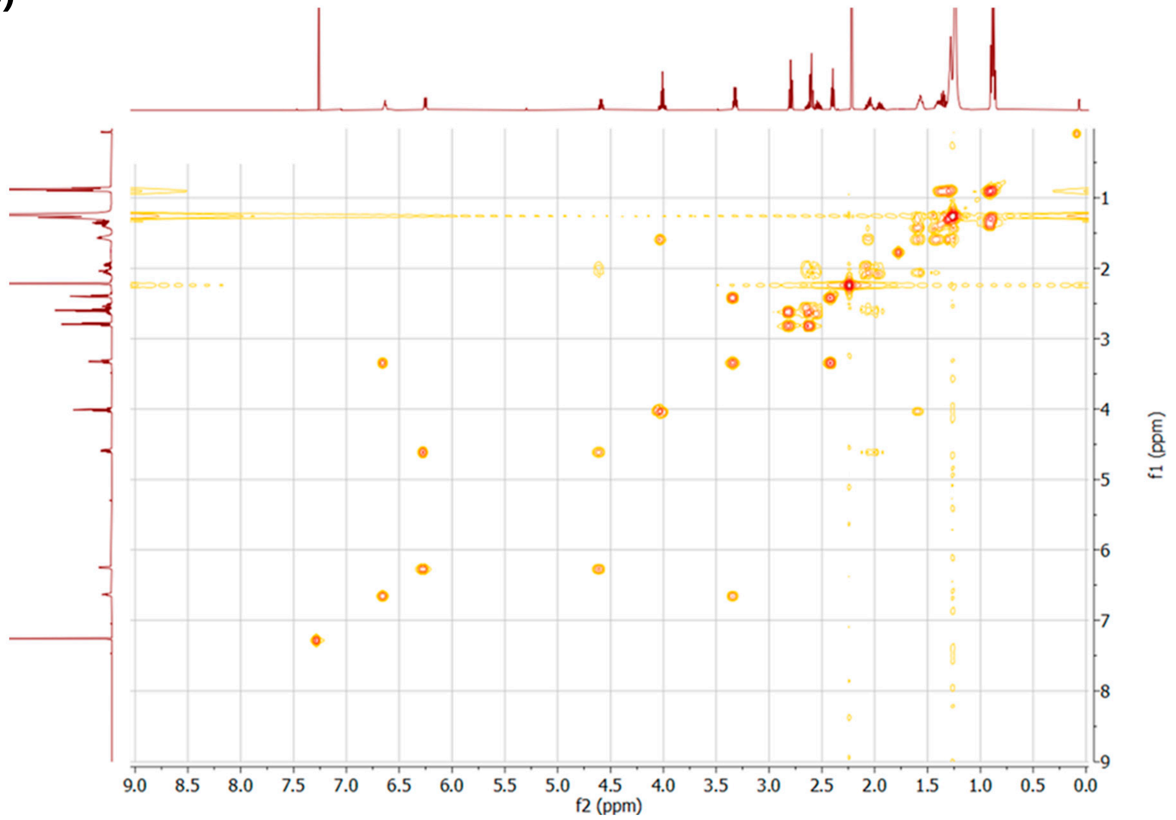

(C)

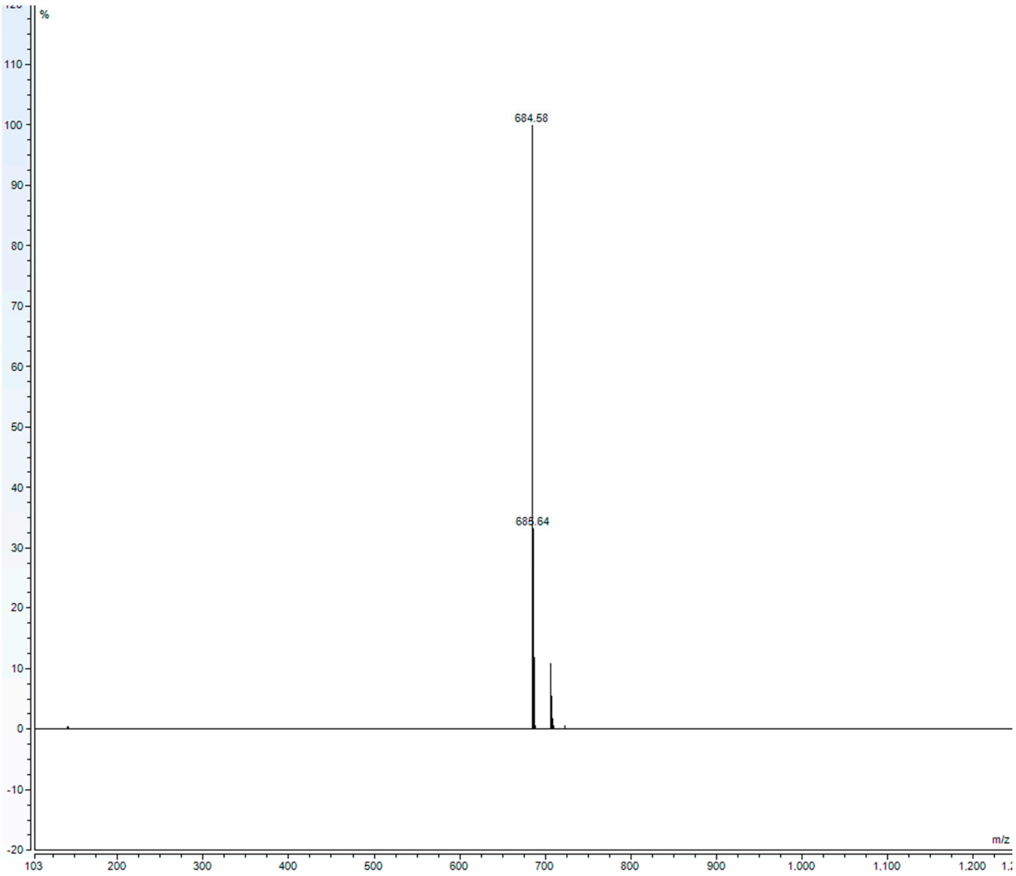

Figure S8. (A) ^{1}H-NMR spectra ($CDCl_3$, 500 MHz) of ionizable lipid CP-LC-0867 with peak assignment. **(B)** 2D COSY spectrum of ionizable lipid CP-LC-0867. **(C)** MS spectra of CP-LC-0867.

(A)

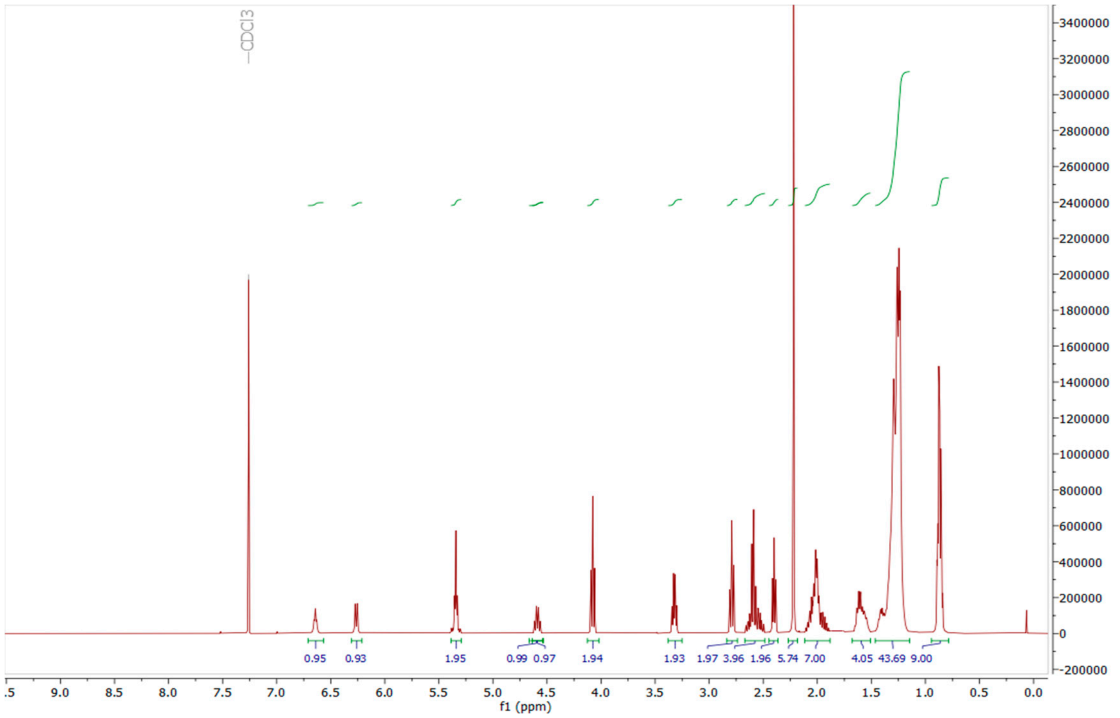
CDCl3
0.95 0.93 1.95 0.99 0.97 1.94 1.93 1.97 3.96 1.96 5.74 7.00 4.05 43.69 9.00
f1 (ppm)

(B)

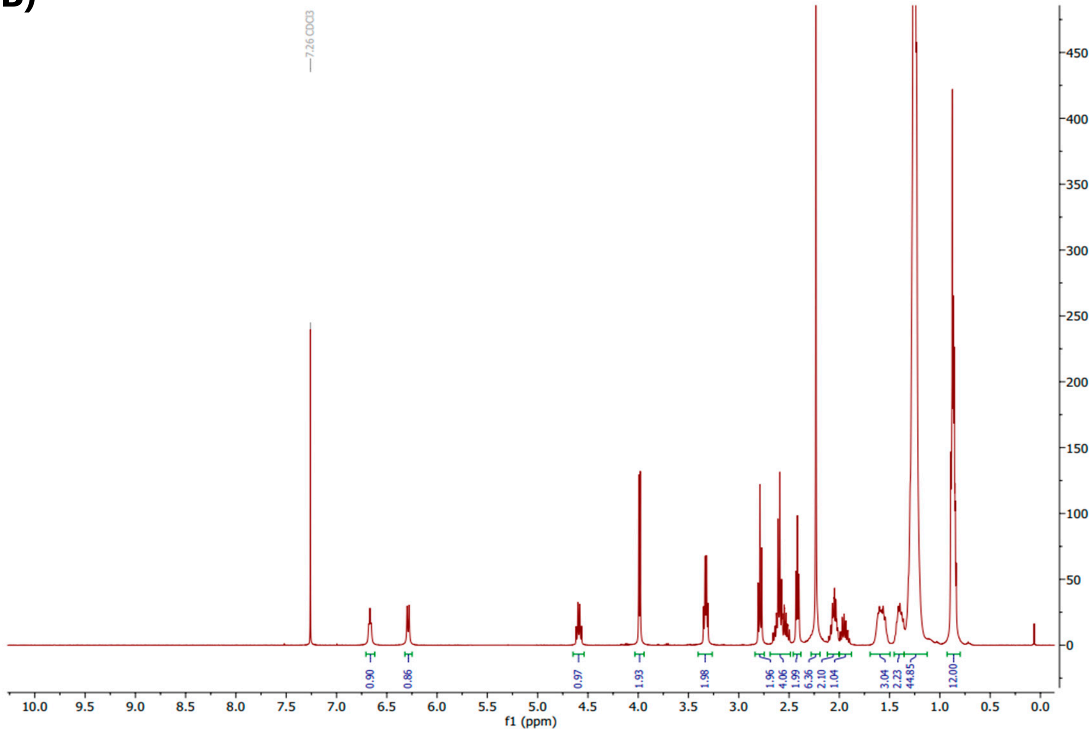
7.26 CDCl3
0.90 0.86 0.97 1.93 1.98 1.96 4.06 1.99 6.36 2.10 1.04 3.04 2.23 44.85 12.00
f1 (ppm)

(C)

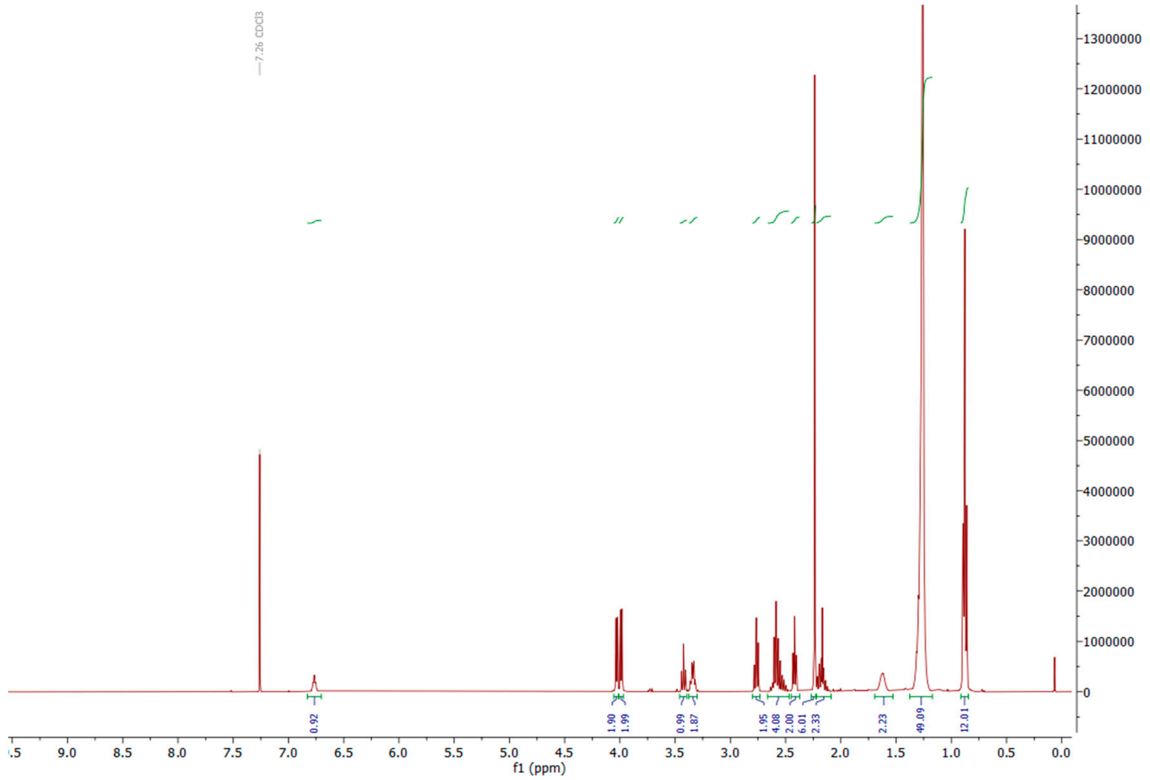

Figure S9. ^{1}H-NMR spectra ($CDCl_3$, 400 MHz) of different ionizable lipids **(A)** CP-LC-0743 **(B)** CP-LC-0729. **(C)** CP-LC-1254.
